# Signification of forkhead box A1 (FOXA1) expression in thyroid cancers

**DOI:** 10.1186/s43046-019-0011-2

**Published:** 2019-12-30

**Authors:** Nabiha Missaoui, Sameh Chouaibi, Sarra Limam, Nozha Mhamdi, Thouraya Zahmoul, Hajer Hamchi, Moncef Mokni, Sihem Hmissa

**Affiliations:** 1 Research Unit UR14ES17, Medicine Faculty, University of Sousse, Sousse, Tunisia; 2 Pathology Department, Farhat Hached University Hospital, Sousse, Tunisia; 3 Faculty of Sciences and Techniques of Sidi Bouzid, Kairouan University, Kairouan, Tunisia; 4 Pathology Department, Sahloul University Hospital, Sousse, Tunisia

**Keywords:** Cancer, Thyroid, FOXA1, C cell, Diagnosis

## Abstract

**Background:**

Forkhead box A1 (FOXA1) plays an important role in several tumors. This study investigated the potential role of FOXA1 expression in thyroid tumors. We conducted a retrospective study of 110 thyroid lesions and tumors diagnosed during 1995–2018. The expression of FOXA1 was analyzed by immunohistochemistry on archival material.

**Results:**

No FOXA1 immunostaining was observed in all cases of Graves’ disease, Hashimoto’s disease, multi-nodular goiter, and adenoma. FOXA1 expression was absent as well in all papillary and follicular carcinomas, Hurthle cell carcinoma, and undifferentiated sarcoma. Only three anaplastic carcinomas exhibited focally FOXA1 staining. However, FOXA1 was expressed in all medullary carcinomas. No significant correlation was found with all clinicopathological features (*p* > 0.05 for all). The pattern of FOXA1 staining was similar to that of calcitonin and chromogranin A (*p* = 0.04 and *p* = 0.003, respectively).

**Conclusions:**

FOXA1 is expressed mostly in all medullary thyroid carcinomas. Hence, FOXA1 could serve as an additional marker for refining the diagnosis of medullary thyroid carcinoma.

## Background

Thyroid cancer is the most common cancer of the endocrine glands, accounting for 1% of all cancers worldwide [1]. In Tunisia, the incidence of thyroid cancer is 2.3/100,000 [2]. Malignant tumors of thyroid follicular cells were classified into well-differentiated, including papillary and follicular carcinomas, and undifferentiated or anaplastic carcinomas [3]. Although the majority of well-differentiated carcinomas are indolent, anaplastic carcinomas are the most aggressive tumors with a median survival time of 6 months [4]. Neither chemotherapy nor radiotherapy seems efficient in improving patient survival time [5]. An alternative treatment for this aggressive malignancy is required.

Medullary carcinoma is the third thyroid malignant tumor originated from the parafollicular cells or C cells, which are the primary site of synthesis and storage of the hormone calcitonin, leading frequently to paraneoplastic syndrome [6]. Almost 25% of medullary thyroid carcinoma cases are inherited disease, involving RET proto-oncogene mutations [7]. As the clinical management of patients diagnosed with medullary carcinomas differs significantly from that of patients with papillary or follicular carcinomas [5], the histopathological diagnosis has important clinical implications [6, 7]. Furthermore, there is a large variety of cellular morphologies and architectural models of medullary carcinoma, leading probably to a diagnostic dilemma [8]. The histopathological diagnosis approval depends certainly on immunohistochemistry data, regarding the expression of calcitonin, carcinoembryonic antigen (CEA), chromogranin A, and other neuroendocrine markers. These carcinomas are positive for calcitonin, with a rate ranging from 93 to 100%. Since the calcitonin and CEA immunostaining could be variable in extent and intensity [9, 10], a more specific marker could improve the diagnosis of these aggressive malignancies and the clinical management of affected patients.

Forkhead box A1 (FOXA1) is a transcription factor belonging to the forkhead box family of DNA-binding proteins [11, 12]. FOXA factors are involved in the development of endoderm-derived organs, including the pancreas, lung, liver, and prostate [12, 13]. FOXA1, also known as hepatocyte nuclear factor 3-alpha (HNF-3A) encoded by the FOXA1 gene, regulates the expression of several genes involved in the development of multiple tissues, such as the mammary gland, lungs, midbrain, and liver [13, 14]. FOXA1 has been shown to open closed chromatin and facilitates the recruitment of other transcription factors [15, 16]. In addition, FOXA1 is reported to participate in diverse human diseases and be involved in the oncogenesis and progression of several tumors such as gliomas, breast, stomach, lung, ovarian, and esophageal cancers [17–20]. In the thyroid tumorigenesis, the potential role of FOXA1 remains not well-elucidated [9, 21]. Nevertheless, FOXA1 expression has been reported in medullary [9] and anaplastic thyroid carcinomas [9, 21]. Further investigations into the molecular mechanisms involved in the development and progression of thyroid cancers are indispensable for a successful management of this cancer.

In this work, we investigated the expression of FOXA1 protein in thyroid lesions and tumors, utilizing whole slide immunohistochemistry. Then, we evaluated the signification of FOXA1 expression in the diagnosis and the prognosis of these tumors.

## Methods

### Tissue samples

We carried out a retrospective study of 110 primary tumors and lesions received in our Pathology Department during 1995–2018. This study was approved by the local Human Ethics Committee of our University Hospital, and it conformed to the provisions of the Declaration of Helsinki.

All hematoxylin and eosin-stained slides were reviewed by 2 pathologists (SH and MM), and the histologic diagnosis was confirmed according to the World Health Organization (WHO) classification, 2017 [22]. The cases studied were distributed into the following groups: 5 Graves’ diseases, 5 Hashimoto’s diseases, 10 multi-nodular goiters, 20 adenomas, and 70 cancers, including 19 papillary carcinomas, 16 follicular carcinomas, 18 medullary carcinomas, 15 anaplastic carcinomas, 1 Hurthle cell carcinoma, and 1 undifferentiated sarcoma.

Samples were selected based on the availability of paraffin-embedded tissue specimens for histological and immunohistochemical analyses. All tissues had been routinely fixed in 10% buffered formalin and paraffin-embedded. One or two tissue blocks were selected to confirm that diagnostic tissue as originally reported was adequately represented in the remaining tissue blocks.

### Clinicopathological features

The collection of clinicopathological data was conducted using patient clinical records from our Pathology Department. Age at diagnosis, gender, histological type, tumor size, and stage were recorded.

### Patient outcome

For patients diagnosed with medullary thyroid carcinoma, outcomes were collected.

### Immunohistochemistry

Immunohistochemistry was performed on sections with a thickness of 4 μm. After dewaxing and rehydration, the antigen unmasking was carried out in a citrate buffer at 95 °C for 40 min. The endogenous peroxidase activity was blocked by 3% hydrogen peroxide. The slides were then incubated with the primary antibody at room temperature (20–25 °C) for 30 min (Table 1). The revelation was made by the Envision+ Dual Link System HRP kit (Dako, code K4063). Diaminobenzidine was used as the chromogen for immunostaining. Finally, the sections were counterstained with hematoxylin and mounted. Appropriate positive controls were performed according to the manufacturer’s instruction. Negative controls were obtained by substitution of the primary antibody by phosphate-buffered saline. Tissue from benign prostate was used as a positive control for FOXA1 staining. Images were captured by the microscopic digital camera Olympus system. Immunohistochemistry evaluation was independently performed by two pathologists. The immunoexpression pattern was scored semi-quantitatively by evaluating the staining extent and was graded as previously described [9].

**Table 1:** Immunohistochemistry conditions and evaluation

Expression	Clone	Provenance	Dilution	Retrieval solution
FOXA1	#654126	RD Systems	1:50	Citrate 0.01 M, pH 6.0
Calcitonin	RbPOLY	Dako	1:200	Citrate 0.01 M, pH 6.0
Chromogranin A	SH7	Dako	1:50	Citrate 0.01 M, pH 9.0

### Statistical analysis

Statistical analysis was performed using the Statistical Package for Social Science (SPSS) software version 19.0 (IBM Corp., Armonk, NY, USA). Probability values (*p*) of 0.05 or less were considered statistically significant.

The relation between the FOXA1 immunohistochemical findings and histopathological type of the thyroid lesion was evaluated using the chi-square test or Fisher’s exact test when appropriate. Additionally, in medullary thyroid cancer, the relationships of FOXA1 expression with clinicopathological features as well as patient outcomes were estimated by the chi-square test or Fisher’s exact test as suitable.

## Results

The patient’ age at diagnosis ranged between 8 and 88 years with a mean age of 54.3 years. There were 85 women and 25 men (sex ratio, 0.29). Tumor size varied from 3 to 100 mm. Overall, 62.9% of cancer cases were diagnosed at a localized stage (I and II), and the remaining samples (37.1%) were diagnosed at an advanced stage (III and IV).

**Fig. 1:**
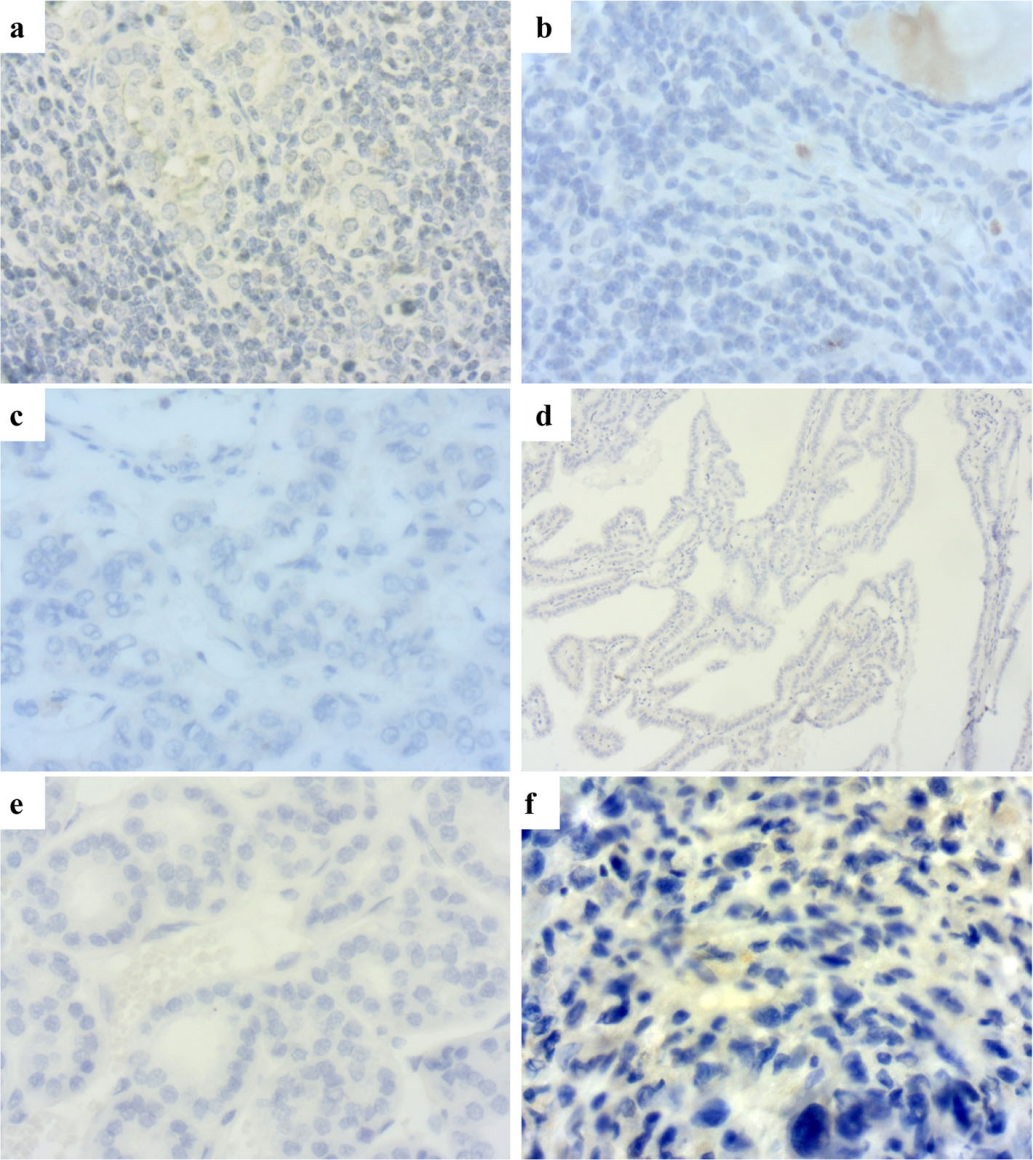
No FOXA1 expression in **a-c** benign tumors (Mx400), **d** papillary carcinoma (Mx200), **e** follicular carcinoma (Mx400), and **f** undifferentiated sarcoma (Mx400)

FOXA1 immunohistochemical findings were summarized in Table 2. No expression of FOXA1 was observed in all cases of Hashimoto’s thyroiditis, Graves’ disease, and multi-nodular goiters (Fig. 1a, b). In addition, FOXA1 expression was not detected in all follicular adenomas as well as Hurthle cell adenoma and hyalinizing trabecular adenoma (Fig. 1c).

**Table 2:** FOXA1 immunostaining findings in thyroid lesions and tumors

Histopathological type	Number	FOXA1-positive
Graves’ disease	5	0 (0%)
Hashimoto’s disease	5	0 (0%)
Multi-nodular goiter	10	0 (0%)
Adenoma	20	0 (0%)
Follicular adenoma	18	0 (0%)
Hyalinizing trabecular adenoma	1	0 (0%)
Hurthle cell adenoma	1	0 (0%)
Cancers	70	21 (30%)
Papillary carcinoma	19	0 (0%)
Follicular carcinoma	16	0 (0%)
Medullary carcinoma	18	18 (100%)
Anaplastic carcinoma	15	3 (20%)
Hurthle cell carcinoma	1	0 (0%)
Undifferentiated sarcoma	1	0 (0%)

**Fig. 2:**
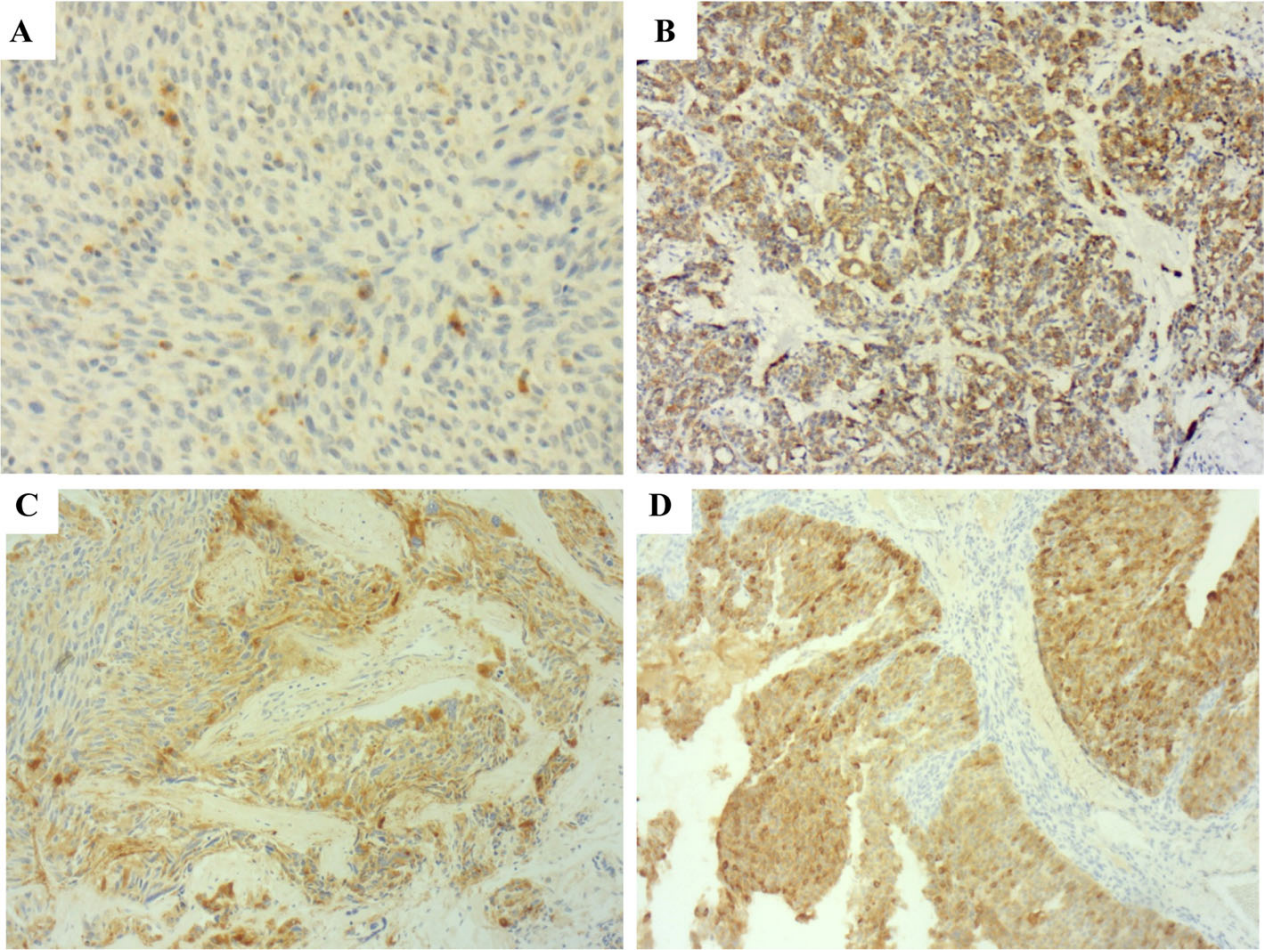
Immunohistochemical findings in anaplastic and medullary carcinomas. **a** Focal expression of FOXA1 in anaplastic carcinoma (Mx200). Positive expression of FOXA1 (**b**), calcitonin (**c**), and chromogranin A (**d**) in medullary carcinomas (Mx200)

Furthermore, all differentiated carcinomas did not exhibit any expression of FOXA1 (Fig. 1d, e). No FOXA1 expression was observed also in Hurthle cell carcinoma and undifferentiated sarcoma. However, only three anaplastic carcinomas expressed focally FOXA1 (score 1+, Fig. 2a).

The expression of FOXA1 was detected in all medullary carcinoma cases (Fig. 2b). The expression score was 4+ in nine medullary carcinomas and 3+ in the remaining cases (Table 3). No significant correlation was found between FOXA1 expression and clinicopathological features of these carcinomas, including patient age (*p* = 0.3), gender (*p* = 0.7), tumor size (*p* = 0.7), and stage (*p* = 0.4).

**Table 3:** Characteristics of medullary thyroid carcinomas

Clinicopathological features					Patient outcome			Immunostaining results		
Case	Gender	Age	Size (cm)	Tumor stage	Recurrence	Recurrence type	Interval time to recurrence	Calcitonin	Chromogranin A	FOXA1
1	F	73	3.8	II	Yes	Loco-regional	2 years	4+	4+	4+
2	F	34	3	II	Patient lost	–	–	4+	4+	4+
3	M	53	1	I	No	–	–	4+	3+	3+
4	M	56	0.3	I	No	–	–	3+	3+	3+
5	M	49	1.5	II	No	–	–	4+	3+	4+
6	F	66	3	II	Yes	Loco-regional	2 years	4+	4+	4+
7	M	65	5	IV	Yes	Distant	6 months	3+	4+	4+
8	F	70	2	II	No	–	–	3+	3+	3+
9	F	42	4.5	III	Yes	Loco-regional	18 months	3+	3+	4+
10	F	53	1	I	No	–	–	2+	3+	3+
11	M	57	5.5	IV	No	–	–	4+	4+	4+
12	F	41	1	I	No	–	–	2+	2+	3+
13	F	65	3	II	No	–	–	4+	3+	3+
14	M	73	4.5	IV	No	–	–	4+	4+	4+
15	F	55	1.2	I	No	–	–	3+	4+	4+
16	F	58	2	II	Patient lost	–	–	3+	3+	3+
17	F	55	2.8	III	No	–	–	3+	3+	3+
18	F	53	2.1	II	No	–	–	3+	3+	3+

All medullary carcinomas exhibited calcitonin and chromogranin A immunostaining (Fig. 2c, d). The expression pattern was scored ≥ 3+ in 88.9% and 94.5% of cases, respectively (Table 3). Although all medullary carcinoma cases expressed FOXA1 with score 3+, the pattern of FOXA1 staining was similar to that of calcitonin and chromogranin A (*p* = 0.04 and *p* = 0.003, respectively).

For patients diagnosed with medullary carcinoma, the follow-up data were available for 16 patients and 2 patients were lost during treatment. The follow-up period ranged from 4 to 96 months with a median of 24 months. Overall, tumor recurrence occurred in 4 subjects (Table 3). Overall, no significant relation was identified between FOXA1 expression and tumor recurrence all over (*p* = 0.08) and loco-regional tumor recurrence (*p* = 0.2) (Table 3).

## Discussion

In this study, FOXA1 expression was investigated in thyroid lesions and tumors using whole immunohistochemical sections. No expression was detected in all inflammatory diseases, all multi-nodular goiters, and all adenomas, including follicular, Hurthle cell, and hyalinizing trabecular adenomas. Previously, Nucera et al. [21] reported as well that there was no expression of FOXA1 in all 30 follicular adenomas, 58 nodular hyperplasias, 8 lymphocyte thyroiditis, and 6 Graves’ diseases. More recently, no FOXA1 immunostaining was observed by Nonaka [9] in all benign thyroid lesions, including 10 nodular hyperplasias, 10 Hashimoto thyroiditis, and 10 Graves’ diseases.

Moreover, no FOXA1 immunostaining was detected in all differentiated thyroid carcinomas, including the 19 papillary carcinomas and 16 follicular carcinomas. Similarly, Nucera et al. [21] showed that the 60 differentiated carcinomas (48 papillary carcinomas and 12 follicular carcinomas) exhibited no specific FOXA1 expression. Furthermore, Nonaka [9] neglected any FOXA1 staining in 21 papillary carcinomas, 27 follicular, and 13 low-grade carcinomas. In addition, no FOXA1 expression was found in 15 Hurthle cell carcinomas of the thyroid [9]. Herein, the only case of Hurthle cell carcinoma did not exhibit any FOXA1 positivity.

The absence of FOXA1 expression in benign lesions as well as in well-differentiated cancers of the thyroid could be explained by the silencing of the *FOXA1* gene, transcriptional repression, or hypermethylation of the *FOXA1* gene. Interestingly, in a very recent study, Chen et al. [23] demonstrated that FOXA1 is a direct target of miR-132 in papillary carcinoma cells. The FOXA1 decrease in thyroid cancer cells significantly inhibits cell proliferation, migration, and invasion, mimicking the suppression effect induced by the overexpression of miR-132 [23]. Restoration of FOXA1 expression partially reversed the suppression effect induced by the miR-132 overexpression. Therefore, the authors claimed that miR-132 acts as a tumor suppressor targeting FOXA1 in thyroid papillary carcinoma [23]. Similarly, in other human diseases, FOXA1 expression has been found to be inhibited by miR-30a-5p and miR-212 in hepatocellular carcinomas [24, 25], by miR-212 in osteosarcomas [26], by miR-194 in non-small cell lung cancers [27], and by miR-431 in necrotizing enterocolitis [28].

The current study included only one case of undifferentiated sarcoma of the thyroid. To the best of our knowledge, there was no previous study analyzing the expression of FOXA1 in mesenchymal tumors of the thyroid. The absence of FOXA1 expression in all tumor cells neglected the involvement of FOXA1 in the development of this mesenchymal tumor. More multicenter studies, using a much larger series of these rare malignancies, will be necessary to further confirm these results.

In our study, only three anaplastic carcinomas exhibited focally FOXA1 immunostaining. By contrast, in the Nucera et al. study [21], FOXA1 expression was reported in 90% of anaplastic carcinomas. The expression was strong in 70% of cases, weak in 20% of cases, and absent in the remaining cases [21]. In addition, the DNA copy number of FOXA1 in the 14q21.1 locus was analyzed by fluorescent in situ hybridization [21]. Nuclear overexpression of FOXA1 was associated with the amplification of the number of FOXA1 DNA copies. Therefore, these researchers suggested the potential role of FOXA1 as an oncogenic transcription factor in thyroid cancer [21]. More recently, Nonaka [9] analyzed also the expression of FOXA1 in a larger series of anaplastic carcinomas. The expression of FOXA1 was detected in 55% of the studied cases, and it was variable in intensity and extent [9]. Furthermore, well-differentiated components were all FOXA1-negative. No correlation was found between the FOXA1 staining and morphological subtype of anaplastic carcinomas [9]. The difference in FOXA1 expression pattern observed between our study and previous reports could be explained partly by technical particularities. The immunohistochemistry on tissue microarray or whole sections and the use of different anti-FOXA1 antibody clones could be at the origin of this discrepancy.

To our knowledge and until the submission of this paper, there were only few previous studies investigating the FOXA1 expression in medullary carcinomas [9, 29]. Nonaka [9] described a diffuse and homogeneously strong FOXA1 nuclear expression in the tumor cells of all 67 medullary carcinomas regardless of cell type, growth pattern, mitotic count, presence of necrosis, and primary or metastatic. As compared to the heterogeneous expression of calcitonin and CEA reported in the same cases, Nonaka [9] considered that FOXA1 expression could serve as a reliable auxiliary marker for the diagnosis of medullary carcinoma of the thyroid. Interestingly, herein, we observed FOXA1 staining in all medullary carcinomas. Although we used a different FOXA1 antibody clone, the immunostaining was scored either 4+ or 3+ in all medullary carcinomas. Furthermore, the pattern of FOXA1 staining was similar to that of calcitonin and chromogranin A. These findings altogether support the usefulness of FOXA1 expression as a potential biomarker refining the diagnosis of medullary thyroid carcinoma. Nevertheless, since there was no significant correlation with all clinicopathological parameters and tumor recurrence, the prognostication role of FOXA1 is limited in these thyroid carcinomas.

In our study, the expression of FOXA1 was detected mainly among medullary carcinomas, supporting its specificity to thyroid C cell tumors. Interestingly, Nonaka [9] showed that all foci of C cell hyperplasia associated with thyroid lesions were diffusely and strongly FOXA1-stained. In addition, recently, the embryonic origin of thyroid C cells in mice and humans has been revised by Johansson et al. [29]. Their study demonstrated that mouse thyroid C cells developed from the pharyngeal endoderm and not from the neural crest. Moreover, propagation of the C cell lineage involved FOXA1 and FOXA2 both together play crucial roles in organogenesis from the foregut endoderm [29]. Additionally, Johansson et al. [29] showed that FOXA1 promotes the growth of neoplastic thyroid C cells. Furthermore, strong FOXA1 and FOXA2 immunostaining was detected in human medullary thyroid carcinomas, including both primary tumor nodules and lymph node metastases [29].

Finally, the current study presented some limitations since it is a retrospective survey of a relatively small number of cancer cases and heterogeneous histological types of thyroid cancer. Thereby, more multicenter studies, using a much larger series of medullary and anaplastic carcinomas, should be conducted to further confirm our findings.

## Conclusion

In summary, since FOXA1 is expressed mostly in all medullary thyroid carcinomas, it could serve as an additional diagnostic marker for medullary carcinoma of the thyroid. Nevertheless, a much larger series of anaplastic carcinomas should be investigated to shed further light on the signification of FOXA1 in these aggressive tumors. Advance genetic and epigenetic studies would be required to better understand the role of FOXA1 in thyroid cancers.

## Data Availability

Not applicable.

## Abbreviations

CEA: Carcinoembryonic antigen
FOXA1: Forkhead box A1
WHO: World Health Organization
